# Benchmarking the Plasmon-Pole and Multipole Approximations in the Yambo Code Using the GW100 Data Set

**DOI:** 10.1021/acs.jctc.6c00239

**Published:** 2026-06-12

**Authors:** M. Bonacci, D. A. Leon, N. Spallanzani, E. Molinari, D. Varsano, A. Ferretti, C. Cardoso

**Affiliations:** † PSI Center for Scientific Computing, Theory and Data, 5232 Villigen PSI, Switzerland; ‡ National Centre for Computational Design and Discovery of Novel Materials (MARVEL), 5232 Villigen PSI, Switzerland; § Istituto Nanoscienze—CNR, S3, Via G. Campi 213/A, 41125 Modena, Italy; ∥ Department of Mechanical Engineering and Technology Management, Norwegian University of Life Sciences, NO- Ås 1432, Norway; ⊥ Dipartimento di Scienze Fisiche, Informatiche e Matematiche, Università di Modena e Reggio Emilia, Modena I-41125, Italy

## Abstract

Verification and validation of electronic structure codes are essential to ensure reliable and reproducible results in computational materials science. Besides the extensive work on density functional theory, in the field of many-body perturbation theory, the GW100 data set represents a key effort to benchmark and validate methods such as the GW approximation. In this work, we assess the numerical accuracy and convergence behavior of the GW implementation in the yambo code using both the Godby–Needs plasmon-pole model (GN-PPA) and the recently introduced multipole approximation (MPA). Quasiparticle energies are compared against GW100 reference data to evaluate the performance, numerical stability, and consistency of these approaches. Our results show that the GN-PPA approach is in very good agreement with full-frequency methods, further improved by the excellent agreement of the MPA scheme, especially when comparing with plane-wave codes.

## Graphical abstract

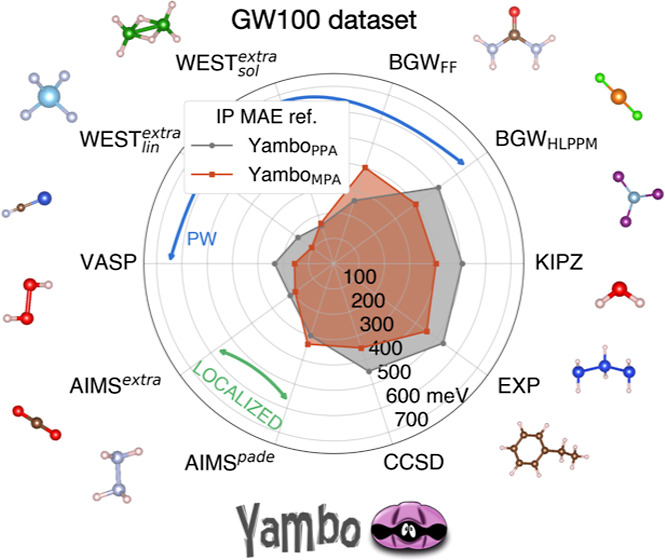

## 1 Introduction

First-principles computational methods have become well-established in the realm of electronic structure and materials science, serving as invaluable tools that offer critical insight and steer experimental investigations, as well as playing a pivotal role in materials discovery and characterization.^1–6^ Once a theoretical framework is defined, such as density functional theory (DFT) or the GW approximation within many-body perturbation theory (MBPT), different numerical implementations should ideally yield equivalent results. In practice, however, algorithmic choices, numerical parameters, and approximations can introduce discrepancies. For this reason, verification (ensuring consistency across codes) and validation (assessing accuracy against experiments) have become key aspects of methodological development. Together, verification and validation (V&V) are essential to establish the reliability, reproducibility, and predictive power of computational techniques.

During the past decade, several systematic comparisons have demonstrated that state-of-the-art DFT codes yield highly consistent results for the ground-state properties of solid-state systems.^7–9^ On the other hand, excited-state properties, such as quasi-particle energy levels of materials calculated with GW in MBPT, have been primarily compared with experiments,^10–16^ while code-to-code V&V studies have emerged more recently.^17–20^ For example, the work by Rangel et al.^17^ compares G_0_W_0_ quasiparticles (QPs) of selected solids (Si, Au, TiO_2_, and ZnO), obtained with three different plane-wave (PW) codes: yambo,^21,22^ berkeleygw^23^ (BGW), and abinit^24^. The comparison shows that, when equivalent convergence parameters, numerical schemes, and pseudopotentials are employed, QP energies agree within 100 meV. Their systematic analysis traced previously reported discrepancies to differences in the treatment of the Coulomb divergence and frequency-integration schemes. By making these aspects consistent across codes, they achieved consistent results even for challenging systems such as rutile TiO_2_ and ZnO.^25–27^

Concerning molecules, early studies have focused on benchmarking different flavors of the GW approximation, including different DFT starting points, with respect to small data sets of experimental results and accurate quantum chemistry calculations based, e.g., on coupled cluster methods.^28–33^ A significant community effort was made to V&V different GW implementations, using the larger GW100 data set, which includes 100 different molecules.^34–40^ This effort is primarily focused on the single-shot G_0_W_0_ calculation of the vertical ionization potential (IP) and electron affinity (EA),^34,36,37^ although results from self-consistent GW schemes have also been reported.^35^ The chosen data set comprises only closed-shell molecules, thereby avoiding the well-known issues presented by open-shell systems.^41^

In terms of both single eigenvalues and statistical deviations (e.g., mean absolute errors), the reported differences between codes using PW and localized basis sets are of the order of 200 meV. The discrepancies are mainly due to (*i*) the size of the adopted basis sets and to (*ii*) the frequency-dependent treatment of the screened Coulomb potential *W* and of the self-energy Σ. Besides the basis sets, the treatment of core and valence electrons can also introduce some differences. Approaches based on pseudopotentials (PPs) reduce the number of explicitly treated electrons with respect to all-electron approaches. Nevertheless, different PP schemes (e.g., ultrasoft vs norm-conserving), the use of nonlinear core corrections (NLCC), or the inclusion of semicore states in valence can significantly affect GW QP energies.^42–44^ Concerning the frequency dependence, the performance of the different plasmon-pole approximation (PPA) models has been addressed in the literature for a few bulk materials.^26,45,46^ For the GW100 data set, however, only the Hybertsen and Louie (HL) model^47^ has been benchmarked against full-frequency (FF) results, while a benchmark for the largely adopted Godby and Needs (GN) model^48^ is still lacking.

In this work, we provide the results for IP and EA of all 100 molecules of the GW100 set as computed within the PW code yambo, using two different frequency dependence treatments: the Godby–Needs PPA (GN-PPA)^48^ model and the recently developed multipole approximation (MPA).^49,50^ Our results show that GN-PPA, at variance with Hybertsen–Louie generalized PPA (HL-PPA), is in good agreement with GW FF data obtained from other PW codes, with an average deviation of only 190 meV. MPA further reduces the deviation to 143 meV, which is comparable to the relative deviation among other FF approaches.

This paper is organized as follows. In Section 2, we provide details about the GW100 set, the adopted GW methodology and implementation, the numerical parameters used in the simulations, and the techniques employed to automate the calculations. In Section 3, we present and discuss the IP and EA results for the GW100 data set, obtained with both GN-PPA and MPA. Our conclusions are drawn in Section 4.

## 2 Computational Approach

### 2.1 The GW100 Data set

The GW100 set comprises a collection of 100 closed-shell molecules,^34^ selected to include a wide range of IP energies (4–25 eV) as well as a variety of chemical bond arrangements. The set includes carbon-based covalently bonded compounds like C_2_H_2_, C_2_H_4_, C_2_H_6_, as well as ionically bonded molecules such as the alkaline metal halide LiF. There are also molecules with metal atoms, such as Ag_2_, Li_2_, K_2_, or small metal clusters (Na_4_, Na_6_), and common molecules such as water and carbon mono- and dioxide.

All molecular structures considered in this study have been taken from the official GW100 repository,^40^ derived either from experiments or optimized using DFT-PBE with the def2-QZVP basis set.^34^ For the cases of CH_2_CHBr and C_6_H_5_OH, for which there is more than one reported structure, we have considered the most recent ones, as described in ref 36. Following previous GW100 benchmarks, the present calculations employ a single-shot *G*_0_ *W*_0_ scheme based on KS-DFT eigenvalues and eigenvectors obtained with the PBE functional,^51^ hereafter denoted as *G*_0_ *W*_0_@PBE. This choice allows us to bypass the well-known dependence of *G*_0_ *W*_0_ results on the starting point,^43,52,53^ and to focus the discussion on the different GW implementations and underlying numerical approximations.

### 2.2 DFT Setup

Even with the same exchange–correlation functional and molecular geometries, the outcome of *G*_0_ *W*_0_ calculations depends on several technical aspects of the underlying DFT setup. These include the adopted basis set, the treatment of core electrons (e.g., via PPs), the definition of the simulation cell and boundary conditions, and the treatment of the long-range Coulomb interaction between periodic replicas. In Table 1, we summarize the methodologies and approximations implemented in the codes benchmarked with the GW100 data set,^34,37^ as well as those implemented in yambo and used in the present work (see details below).

**Table 1:** Basis set and Approximations Used in Previous GW100 Benchmarks and in the Present Work^a^

code	basis set	PP	empty states	χ-basis	Σ_*c*_-integration	QP solutions	refs.
TM^54^	def2-QZVP	AE	all	LO	FA^55^	largest weight	^34^
FHI-aims^56^	def2-QZVP*	AE	all	LO	AC^56,57^	iterative	^34^
BGW^23^	PW	TM NC^58^	truncated^59^	PW	HL-PPA^47^/FF^60^	lin./graphical	^34^
VASP^61^	PAW*	PAW	truncated	PW*	AC^62^	linearized	^36^
WEST^63^	PW	ONCV	none^63,64^	PDEP*^65^	CD^66^	secant/lin.	^37^
yambo^21,22^	PW	ONCV	truncated^67^	PW	GN-PPA^48^/MPA^49^	linearized	this work

a The codes TM and FHI-aims make use of the resolution-of-identity (RI) technique to compute the four-center Coulomb integrals.^34^ In the column basis set, * indicates results obtained after extrapolation. In the column *PP*, we list the approach used for core and valence electrons: all electron (AE), Troullier–Martins norm conserving pseudopotentials (TM NC), projector augmented waves (PAW), and optimized norm-conserving Vanderbilt pseudopotentials (ONCV). In the column Σ_*c*_ *-*integration, the acronyms refer to the frequency integration methods used for the self-energy: fully-analytic (FA), analytic continuation (AC), contour deformation (CD), Godby–Needs plasmon-pole model (GN-PPA), and multipole approximation (MPA).

The GW100 data were originally obtained using a variety of basis sets, including projector augmented waves (PAW),^68,69^ linear augmented plane waves (LAPW),^70^ linearized muffin-tin orbitals (LMTO),^71^ and local orbital (LO) basis sets. For a detailed comparison of IPs and EAs obtained using DFT-PBE with different basis sets, we refer the reader to ref 37. In this work, the Kohn–Sham (KS) wave functions and charge densities are expanded in plane waves (PWs) under periodic boundary conditions (PBCs), as implemented in the quantum espresso simulation package.^72,73^

The treatment of core and valence electrons varies across different codes. Approaches based on PPs reduce the number of explicitly treated electrons, simplifying the calculations while retaining accuracy for valence states. Nevertheless, different PP schemes (ultrasoft vs norm-conserving), the use of NLCC, or the inclusion of semicore states in valence can significantly affect GW QP energies.^42–44^ Here, we employ optimized norm-conserving Vanderbilt PPs from the SG15 library,^74,75^ without NLCC.^76^ Details of the electronic configurations, including the semicore states treated as valence, are provided in the Supporting Information.^75^

Each molecule is placed in a supercell with sufficient vacuum to suppress spurious interactions between replicas. To reduce the required vacuum spacing and the associated computational cost, the Martyna–Tuckerman method^77^ is adopted to correct the error introduced by the periodic replica of the system. Choosing the lattice geometry in order to minimize the supercell volume is also essential for both numerical accuracy and computational efficiency. Previous GW100 studies^37^ used simple cubic (SC) supercells with lattice parameters up to *a* = 25 Å. In the present work, we adopt a face-centered cubic (FCC) geometry with lattice parameter *a* = 13 Å, which reduces the supercell volume by a factor of $\sqrt{2}$ relative to the corresponding SC configuration. Tests performed on two molecular systems, with 10 and 56 atoms (the latter being the largest molecule in the GW100 set), show a residual IP/EA error of ∼25 and ∼180 meV, respectively (see Sec. II of the Supporting Information^78^).

At the GW level, a real-space truncation of the Coulomb interaction is applied to both the screening and self-energy terms.^79^ This spherical Coulomb cutoff, with a diameter of approximately 12 Å (i.e., 1 Å smaller than the lattice parameter), analytically removes the divergence of the long-range Coulomb interaction *v*(***q*** → 0) and ensures rapid convergence with respect to the size of the supercell.

### 2.3 The QP Equation

Within the MBPT framework, QP energies are usually obtained perturbatively from DFT, as the solutions of the QP equation

$$\varepsilon_{nk}^{QP}=\varepsilon_{nk}+\left\langle nk\left|\Sigma \left(\varepsilon_{nk}^{QP}\right)-v_{xc}\right|nk\right\rangle$$ 1

where Σ is the electron self-energy, {ε_*n* **k**_, |*n* **k**⟩} are the reference Kohn–Sham (KS) eigenvalues and wave functions, and *v*_*xc*_ is the corresponding exchange–correlation potential. eq 1 originates from a nonlinear eigenvalue problem,^80,81^ where the self-energy has been assumed to be diagonal in the reference basis. This reduces the problem to a scalar but still nonlinear equation for each state, possibly leading to multiple solutions. To simplify the problem, one can linearize the self-energy as the first-order term of the Taylor expansion around ε_*n* **k**_, yielding

$$\varepsilon_{nk}^{QP}=\varepsilon_{nk}+Z_{nk}\left\langle nk\left|\Sigma \left(\varepsilon_{nk}\right)-v_{xc}\right|nk\right\rangle$$ 2

with the renormalization factor

$$Z_{nk}={\left[1-{\left\langle nk|\frac{\partial \Sigma \left(\omega \right)}{\partial \omega }|nk\right\rangle |}_{\omega =\varepsilon_{nk}}\right]}^{-1}$$ 3

eqs 1 or 2 can be solved numerically by using iterative root-finding methods. Multiple solutions have been reported, for instance, for some molecules in the GW100 benchmark.^34^ When both the real and imaginary parts of Σ are considered, the different solutions correspond to the poles of the interacting Green’s function *G* and can be assigned one to the main QP excitation and the others to satellites. The solutions can also be determined analytically using alternative approaches based on rational representations of Σ, through, for example, the algorithmic inversion method for sum-overpoles (AIM-SOP) reported in refs 82–85 or the MPA-Σ approach reported in ref 86.

In this work, we adopt the linearized form of eq 2, solved using Newton’s method as implemented in the yambo code. This approach assumes that Σ varies smoothly around the QP energy and therefore neglects secondary (satellite) solutions.

### 2.4 GW Self-Energy Evaluation

The state-of-the-art method for evaluating the self-energy Σ is the one-shot *G*_0_ *W*_0_ approximation. In this approximation, Σ is given by a frequency convolution of the noninteracting KS Green’s function *G*_0_ and the screened Coulomb potential *W*_0_

4 $$\Sigma \left(\omega \right)=\frac{i}{2\pi }\int d\omega^{\prime }e^{i\omega^{\prime }0^{+}}G_{0}\left(\omega +\omega^{\prime }\right)W_{0}\left(\omega^{\prime }\right)$$

In the expression above, *W*_0_ is usually evaluated at the random-phase approximation (RPA) level and is typically split into two terms: the bare Coulomb interaction *v*, which is static, and the correlation part *W* ^*c*^ ≡ *W*_0_ – *v*, which contains all the frequency dependence. This, in turn, leads to a decomposition of the self-energy into exchange and correlation terms, Σ­(ω) = Σ^*x*^ + Σ^*c*^(ω).

The dynamical nature of the screened interaction *W* distinguishes the GW approximation from static theories like Hartree–Fock. A number of different strategies have been proposed to evaluate the frequency integral in eq 4, ranging from full-analytic^55^ (FA) to full-frequency real-axis integration^60^ (FF), analytic continuation^87^ (AC), contour deformation (CD),^66^ and multipole approaches (MPA).^49,50^ The earliest and simplest approach is the so-called PPA.^47,48^ Within PPA, the frequency dependence of *W* ^*c*^ is simplified as a symmetric single pole function for each matrix element **GG**′ and transferred momentum **q**

5 $$W_{GG^{\prime }}^{cPPA}\left(q,\omega \right)=\frac{2R_{qGG^{\prime }}\Omega_{qGG^{\prime }}}{\omega^{2}-\Omega_{qGG^{\prime }}^{2}}$$

Among the different flavors of PPA,^26^ in this work, we adopt the one proposed by Godby and Needs (GN-PPA),^48^ which interpolates *W* ^*c*^ from the values computed at two points, ω = 0 and ω = *iE*_PPA_. Here, we set *E*_PPA_ = 30 eV, after having tested a subset of molecules by varying the sampling energy between 25 and 30 eV, without significant changes in the results. The HL-PPA^47^ model follows a different approach and, in addition to evaluating the screening at zero frequency, imposes the fulfillment of Johnson’s frequency sum rule (*f*-sum rule).^88^ Although such a property can be mapped with the GN-PPA model, by increasing *E*_PPA_ to infinity,^49^ the results of the standard GN-PPA and HL-PPA may differ significantly. For a detailed comparison of different PPA models, see, e.g., refs 26, 45, and 46 .

Besides GN-PPA, we also adopt the MPA method,^49,50^ which generalizes eq 5 to multiple complex poles

6 $$W_{GG^{\prime }}^{cMPA}\left(q,\omega \right)=\sum_{p}^{n_{p}}\frac{2R_{pqGG^{\prime }}\Omega_{pqGG^{\prime }}}{\omega^{2}-\Omega_{pqGG^{\prime }}^{2}}$$

where *n*_*p*_ is typically around 10. Making use of the Lehmann representation for *G*_0_ in a Bloch PW basis set, and the MPA model for *W* ^*c*^, the diagonal matrix elements of the correlation self-energy are integrated analytically and can be expressed as

7 $$\Sigma_{nk}^{cMPA}\left(\omega \right)=\sum_{m}\sum_{GG^{\prime }}\sum_{p}^{n_{p}}\int \frac{dq}{{\left(2\pi \right)}^{3}}S_{pGG^{\prime }}^{nm}\left(k,q\right)\left[\frac{f_{mk-q}^{KS}}{\omega -\varepsilon_{mk-q}^{KS}+\Omega_{pqGG^{\prime }}}+\frac{1-f_{mk-q}^{KS}}{\omega -\varepsilon_{mk-q}^{KS}-\Omega_{pqGG^{\prime }}}\right]$$

where we have defined

$$\begin{array}{lll} S_{pGG^{\prime }}^{nm}\left(k,q\right) & = & -2\rho_{nm}^{KS}\left(k,q,G\right)R_{pqGG^{\prime }}{\rho_{nm}^{KS}}^{*}\left(k,q,G^{\prime }\right)\\ \rho_{nm}^{KS}\left(k,q,G\right) & = & \left\langle nk\left|e^{i\left(q+G\right)\cdot r}\right|mk-q\right\rangle \end{array}$$ 8

For each momentum transfer **q** and each **GG**′ matrix element, poles and residues are obtained through a nonlinear interpolation with frequency points *z*_*i*_ sampled in the complex plane

9 $$\sum_{p=1}^{n_{p}}\frac{2R_{pqGG^{\prime }}\Omega_{pqGG^{\prime }}}{z_{i}^{2}-\Omega_{pqGG^{\prime }}^{2}}=W_{GG^{\prime }}^{c}\left(q,z_{i}\right)$$

where *i* = 1, ..., 2*n*_*p*_ and the set of complex frequencies *z*_*i*_ are conveniently selected according to the double parallel sampling defined in refs 49 and 50 . Such efficient sampling allows the MPA method to achieve FF accuracy with a small number of poles or sampling frequencies, typically 1–2 orders of magnitude fewer than the number of frequency points required to converge FF-RA.

### 2.5 Convergences and Workflow

Accurate GW QP energies require convergence with respect to several computational parameters. In yambo, the most relevant parameters are the number of unoccupied states (*N*_*b*_) that enter the sum over states and the kinetic-energy cutoff (*G*_cut_) defining the number of reciprocal lattice vectors used in the evaluation of the self-energy in eq 7 and the screening matrix in eq 6. Within PPA, convergence with respect to *N*_*b*_ can be accelerated by using the Bruneval–Gonze (BG) terminator,^67^ which replaces the contribution of high-energy states with an effective pole at an energy *E*_GT_ above the highest explicitly included band. For isolated systems, where unoccupied levels are densely spaced, effective *E*_GT_ values are typically smaller than for bulk systems. Tests on LiF for different *E*_GT_ values (Figure 1, left panel) show that 0.25 Ha yields the largest convergence acceleration, and this value is adopted for all molecules.

**Figure 1:**
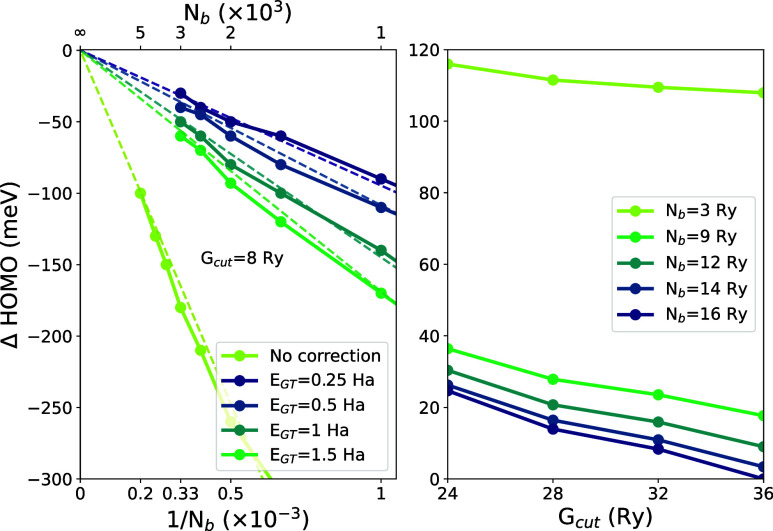
Convergence tests for the LiF molecule. Left panel: Effect of E_GT_ (see text) on the convergence acceleration of the HOMO with respect to empty states summation [the *N*_*b*_ → *∞* (extrapolated) value for the noncorrected case is set to zero in the plot]. Right panel: HOMO energy versus the PW cutoff *G*_cut_ in the screening matrix, for different numbers of empty states, N_*b*_. The result obtained with N_*b*_ = 16 Ry, *G*_cut_ = 36 Ry is set to zero. The values N_*b*_ = 3, 9, 12, 14, and 16 Ry correspond to 1000, 5000, 7000, 9000, and 11000 bands.

As discussed in the literature,^17,26,89^ the parameters *N*_*b*_ and *G*_cut_ are interdependent, as shown in the right panel of Figure 1, where the curves indicate that the QP energies have not yet reached convergence within the accessible range of parameters. In this work, the convergence with respect to these two parameters is assessed simultaneously via extrapolation. Specifically, QP corrections are computed on a two-dimensional grid, with *N*_*b*_ ranging from 1000 to 11000 (3000–13000 for MPA) and *G*_cut_ from 24 to 36 Ry. When possible, an additional point at *G*_cut_ = 38 Ry is included. The extrapolation to infinite cutoff values is obtained by fitting the QP corrections to

10 $$f\left(N_{b},G_{cut}\right)=\left(\frac{A}{N_{b}^{\alpha }}+b\right)\left(\frac{C}{G_{cut}^{\beta }}+d\right)$$

where α and β belong to {1, 2, 3} and, for each molecule, the values that minimize the root-mean-square error are chosen. Similarly to the strategy adopted in refs 90–94 , the fully converged QP energy reads

11 $$E_{extra}^{QP}=f\left(N_{b}\to \infty ,G_{cut}\to \infty \right)=bd$$

All calculations were performed with a fully automated high-throughput (HT) scheme using the aiida-yambo plugin,^89,95^ which is part of the AiiDA workflow ecosystem^96,97^ and implements automated workflows and convergence protocols for MBPT yambo simulations. We note that related efforts have been undertaken by the community in recent years.^98–100^ For each of the 100 molecules, convergence workflows systematically explored the (*N*_*b*_, *G*_cut_) space, resulting in over 9000 QP evaluations across both PPA and MPA schemes. All input and output data are stored in the AiiDA database, ensuring full reproducibility. This work also serves as a large-scale validation of the aiida-yambo workflows. Computational performance statistics are reported in the Supporting Information.^78^

## 3 Results

To benchmark the accuracy of the GN–PPA and MPA implementations of the *G*_0_ *W*_0_ method in yambo, as compared to other codes, we computed the IP and EA energies of the 100 molecules in the GW100 data set. These quantities are evaluated as

12 $$\begin{array}{ll} IP & =E^{vac}-E^{HOMO}\\ EA & =E^{vac}-E^{LUMO} \end{array}$$

where *E* ^vac^ denotes the electrostatic potential in the vacuum region. *E* ^vac^ is evaluated within the Martyna–Tuckerman method,^77^ that automatically sets it to zero by shifting the energy scale (so that *IP* = – *E* ^*HOMO*^ and *EA* = – *E* ^*LUMO*^).

The statistical accuracy of the computed IP energies is characterized by the deviations with respect to the results obtained with the codes we are comparing with. Using standard statistical measures, we present in Table 2 of Sec. [Sec sec3.1] the values for the mean error (ME), the mean absolute error (MAE), the mean absolute relative error (MARE), and the standard deviation (σ). The IP values obtained for each molecule are reported in Table 3, together with reference GW results^34^ from other codes, CCSD data,^101^ and experimental values from the NIST database.^102^ EA results are provided in Table 4 and discussed in Sec. [Sec sec3.2]. While the discussion below focuses primarily on IPs, which provide the most direct and comprehensive benchmark across the different implementations, the same conclusions apply to the EAs, analyzed in Sec. [Sec sec3.2].

**Table 2:** Mean Error (ME), Mean Absolute Error (MAE), Mean Absolute Relative Error (MARE), and Standard deviation (σ) of the IPs Computed with Yambo-GN-PPA and Yambo-MPA with Respect to Reference GW100 Results^34,36,37,101,103^ Obtained with Different Codes and Approximations. We Also Included Experimental (EXP) Data^34,102^

Code	ME (meV)		MAE (meV)		MARE (%)		σ (meV)	
	GN-PPA	MPA	GN-PPA	MPA	GN-PPA	MPA	GN-PPA	MPA
YAMBO_GN‑PPA_	-	77	-	168	-	1.8	-	114
WEST^extra^_lin_	–117	–31	176	108	1.8	1.3	159	121
WEST^extra^_sol_	9	96	159	167	1.8	1.9	165	123
VASP	–44	32	234	155	2.4	1.8	216	199
AIMS^pade^	225	302	299	334	3.4	3.6	320	260
AIMS^extra^	22	119	214	189	2.4	2.1	200	185
BGW_HL‑PPA_	–400	–323	511	400	4.7	3.8	336	321
BGW_FF_	203	387	262	399	2.8	3.9	382	388
KIPZ	–375	–298	507	403	4.8	3.9	398	358
CCSD	–338	–259	447	350	4.1	3.3	374	310
EXP	–255	–175	534	454	5.0	4.3	401	387

**Table 3:** Quasiparticle Ionization Potentials (IP) of the Molecules in the GW100 Data Set as Obtained Within This Work: Y_PPA_ and Y_MPA_ Refer to Yambo Calculations Performed at the PPA and MPA Levels, Respectively; for Completeness, PBE IPs are Also Reported^a^

index	formula	*Y*_MPA_	*Y*_PPA_	WEST_lin_^extra^	WEST_sol_^extra^	VASP	AIMS^pade^	AIMS^extra^	BGW_HL_	BGW_FF_	CCSD	EXP
1	He	23.72	23.33	23.65	23.42	23.62	23.48	23.49	24.1	-	24.51	24.59
2	Ne	20.14	20.06	20.52	20.33	20.36	20.38	20.33	21.35	-	21.32	21.56
3	Ar	15.56	15.34	15.5	15.37	15.42	15.13	15.28	15.94	-	15.54	15.76
4	Kr	13.9	13.79	13.87	13.76	14.03	13.57	13.89	14.0	-	13.94	14.0
5	Xe	13.34	13.52	13.38	13.22	12.22	12.02	-	12.08	-	-	12.13
6	H_2_	16.1	16.05	16.03	15.84	16.06	15.82	15.85	16.23	-	16.4	15.43
7	Li_2_	5.07	5.24	5.19	5.04	5.32	4.99	5.05	5.43	-	5.27	4.73
8	Na_2_	4.89	5.11	5.07	4.98	5.06	4.83	4.88	5.03	-	4.95	4.89
9	Na_4_	3.85	4.09	4.28	4.24	4.23	4.1	4.14	4.34	-	4.22	4.27
10	Na_6_	3.78	4.04	4.42	4.37	4.4	4.24	4.34	4.47	-	4.35	4.12
11	K_2_	3.96	4.3	4.21	4.14	4.24	3.98	4.08	4.02	-	4.06	4.06
12	Rb_2_	3.79	4.14	4.08	4.01	4.14	3.8	-	3.92	-	3.92	3.9
13	N_2_	15.16	14.84	15.08	14.94	15.06	14.89	15.05	15.43	14.72	15.57	15.58
14	P_2_	10.53	10.57	10.48	10.43	10.4	10.21	10.38	10.66	-	10.47	10.62
15	As_2_	9.62	9.7	9.58	9.55	9.62	9.47	9.67	9.67	-	9.78	10.0
16	F_2_	14.89	14.53	15.16	15.0	15.08	14.96	15.1	15.59	14.73	15.71	15.7
17	Cl_2_	11.61	11.45	11.5	11.41	11.4	11.1	11.31	11.85	-	11.41	11.49
18	Br_2_	10.62	10.5	10.52	10.44	10.65	10.22	10.56	10.64	-	10.54	10.51
19	I_2_	10.41	10.61	10.56	10.41	9.59	9.28	-	9.58	-	9.51	9.36
20	CH_4_	14.2	14.03	14.1	13.99	14.14	13.93	14.0	14.28	13.8	14.37	13.6
21	C_2_H_6_	12.63	12.44	12.53	12.44	12.58	12.36	12.46	12.63	12.22	13.04	11.99
22	C_3_H_8_	12.04	11.85	11.92	11.84	11.98	11.79	11.89	12.05	-	12.05	11.51
23	C_4_H_1_0	11.7	11.52	11.48	11.41	11.69	11.49	11.59	11.73	-	11.57	11.09
24	C_2_H_4_	10.52	10.48	10.46	10.39	10.5	10.32	10.4	10.68	10.3	10.67	10.68
25	C_2_H_2_	11.28	11.18	11.18	11.09	11.24	11.02	11.09	11.35	10.97	11.42	11.49
26	C_4_	11.11	10.98	10.97	10.9	10.97	10.78	10.91	11.49	-	11.26	12.54
27	C_3_H_6_	10.78	10.63	10.73	10.67	10.78	10.56	10.65	10.93	-	10.86	10.54
28	C_6_H_6_	9.17	9.1	9.13	9.08	9.16	8.99	9.1	9.21	-	9.29	9.23
29	C_8_H_8_	8.21	8.09	8.2	8.16	8.24	8.06	8.18	8.47	-	8.35	8.43
30	C_5_H_6_	8.56	8.48	8.49	8.44	8.51	8.35	8.45	8.77	-	8.68	8.53
31	C_2_H_3_F	10.4	10.28	10.36	10.29	10.36	10.2	10.32	10.8	10.14	10.55	10.63
32	C_2_H_3_Cl	10.09	9.97	10.0	9.94	10.0	9.76	9.9	10.32	-	10.09	10.2
33	C_2_H_3_Br	9.23	9.17	9.71	9.64	9.83	8.99	9.14	9.42	-	9.27	9.9
34	C_2_H_3_I	9.85	9.9	9.94	9.81	9.36	9.04	-	9.48	-	9.33	9.35
35	CF_4_	15.46	15.13	15.65	15.51	15.53	15.36	15.6	15.96	-	16.3	16.2
36	CCl_4_	11.49	11.35	11.41	11.29	11.31	10.98	11.21	11.77	-	11.56	11.69
37	CBr_4_	10.25	10.15	10.22	10.11	10.38	9.9	10.22	10.4	-	10.46	10.54
38	CI_4_	9.82	10.02	-	-	9.23	8.82	-	9.23	-	9.27	9.1
39	SiH_4_	12.6	12.6	12.55	12.42	12.53	12.31	12.4	12.77	-	12.8	12.3
40	GeH_4_	12.47	12.53	12.44	12.32	12.24	12.02	12.12	12.28	-	12.5	11.34
41	Si_2_H_6_	10.6	10.6	10.58	10.52	10.52	10.31	10.41	10.8	-	10.64	10.53
42	Si_5_H_1_2	8.69	8.7	9.25	9.19	9.19	8.94	9.05	9.45	-	9.27	9.36
43	LiH	7.31	7.55	7.2	6.62	7.2	6.54	6.58	7.85	6.67	7.96	7.9
44	KH	5.55	5.99	5.39	4.97	5.37	4.86	4.99	5.76	-	6.13	8.0
45	BH_3_	13.16	13.07	13.08	12.95	13.09	12.87	12.96	13.28	-	13.28	12.03
46	B_2_H_6_	12.1	12.0	12.03	11.92	12.04	11.84	11.93	12.17	-	12.26	11.9
47	NH_3_	10.46	10.3	10.4	10.18	10.44	10.32	10.39	10.93	-	10.81	10.82
48	HN_3_	10.63	10.35	10.54	10.48	10.56	10.4	10.55	10.96	-	10.68	10.72
49	PH_3_	10.57	10.61	10.51	10.43	10.45	10.27	10.36	10.79	-	10.52	10.59
50	AsH_3_	10.43	10.45	10.4	10.33	10.36	10.12	10.21	10.45	-	10.4	10.58
51	SH_2_	10.43	10.37	10.36	10.23	10.3	10.03	10.13	10.64	-	10.31	10.5
52	FH	15.34	15.03	15.47	15.23	15.38	15.3	15.37	16.24	-	16.03	16.12
53	ClH	12.68	12.58	12.6	12.48	12.51	12.25	12.36	12.97	-	12.59	12.79
54	LiF	10.36	10.32	10.54	10.11	10.45	9.95	10.27	11.84	-	11.32	11.3
55	F_2_Mg	12.65	12.59	12.84	12.46	12.77	12.32	12.5	13.73	12.44	13.71	13.3
56	TiF_4_	14.12	13.95	14.31	-	14.22	13.89	14.07	14.88	-	15.48	13.3
57	AlF_3_	14.45	14.22	14.63	14.4	14.53	14.25	14.48	15.11	-	15.46	15.45
58	BF	10.69	11.08	10.71	10.56	10.67	10.56	10.73	11.49	-	11.09	11.0
59	SF_4_	12.43	12.4	12.41	12.32	12.29	12.12	12.38	12.79	-	12.59	11.69
60	BrK	8.04	8.1	7.96	-	8.04	7.3	7.57	7.99	-	8.13	8.82
61	GaCl	10.35	10.29	10.27	10.19	9.99	9.55	9.74	10.24	-	9.77	10.07
62	NaCl	8.98	9.02	-	-	8.76	8.1	8.43	9.6	-	9.03	9.8
63	MgCl_2_	11.46	11.45	11.47	11.25	11.41	10.99	11.2	11.98	-	11.66	11.8
64	AlI_3_	10.12	10.3	10.59	10.31	9.69	9.32	-	9.67	-	9.82	9.66
65	BN	11.6	11.34	-	-	10.61	11.03	11.15	12.19	9.68	11.89	11.5
66	NCH	13.5	13.3	13.41	13.22	13.43	13.21	13.32	13.87	-	13.87	13.61
67	PN	11.51	11.28	11.43	11.26	11.41	11.14	11.29	12.13	-	11.74	11.88
68	H_2_NNH_2_	9.48	9.21	9.42	9.27	9.45	9.28	9.37	9.78	9.1	9.72	8.98
69	H_2_CO	10.57	10.24	10.56	10.41	10.57	10.33	10.46	11.02	-	10.84	10.88
70	CH_4_O	10.64	10.39	10.74	10.6	10.72	10.56	10.67	11.14	-	11.04	10.96
71	C_2_H_6_O	10.36	10.0	10.36	10.21	10.33	10.16	10.27	10.57	-	10.68	10.64
72	C_2_H_4_O	9.84	9.46	9.81	9.61	9.8	9.55	9.66	10.16	9.43	10.21	10.24
73	C_4_H_1_0O	9.46	9.13	9.52	9.4	9.52	9.32	9.42	9.7	-	9.82	9.61
74	CH_2_O_2_	10.87	10.72	11.01	10.82	10.98	10.73	10.87	11.39	-	11.42	11.5
75	HOOH	11.18	10.79	11.16	11.0	11.12	10.99	11.1	11.58	10.82	11.59	11.7
76	H_2_O	12.08	11.81	12.09	11.87	12.05	11.97	12.05	12.75	11.68	12.56	12.62
77	CO_2_	13.45	13.1	13.46	13.37	13.44	13.25	13.46	13.81	13.17	13.71	13.77
78	CS_2_	10.15	10.09	10.1	10.05	10.01	9.75	9.95	10.37	-	9.98	10.09
79	OCS	11.26	11.15	-	-	11.13	10.91	11.11	11.49	11.02	11.17	11.19
80	OCSe	10.49	10.45	10.43	10.37	10.5	10.2	10.43	10.55	-	10.78	10.37
81	CO	13.81	13.77	13.79	13.66	13.76	13.57	13.71	14.33	-	14.21	14.01
82	O_3_	12.18	11.84	-	-	12.07	11.39	11.49	13.05	12.0	12.55	12.73
83	SO_2_	12.08	11.81	12.08	11.96	12.04	11.82	12.06	12.55	-	13.49	12.5
84	BeO	9.57	9.57	-	-	9.5	8.58	8.6	10.66	9.68	9.94	10.1
85	MgO	7.34	7.32	-	-	7.1	6.68	6.75	8.51	7.08	7.49	8.76
86	C_7_H_8_	8.69	8.65	8.75	8.71	8.79	8.61	8.72	8.97	-	8.9	8.82
87	C_8_H_1_0	8.61	8.52	8.7	8.66	8.73	8.55	8.66	8.92	-	8.85	8.77
88	C_6_F_6_	9.61	9.46	9.7	9.65	9.69	9.49	9.74	10.04	-	9.93	10.2
89	C_6_H_5_OH	8.4	8.36	8.42	8.37	8.43	8.37	8.51	8.72	-	8.7	8.75
90	C_6_H_5_NH_2_	7.79	7.64	7.8	7.73	7.84	7.64	7.78	7.98	-	7.99	8.05
91	C_5_H_5_N	9.32	9.1	9.28	9.13	9.31	9.04	9.17	9.5	-	9.66	9.66
92	C_5_H_5_N_5_O	7.71	7.54	7.86	7.82	8.18	7.69	7.87	7.92	-	8.03	8.24
93	C_5_H_5_N_5_	8.08	7.9	8.14	8.09	8.18	7.98	8.15	8.35	-	8.33	8.48
94	C_4_H_5_N_3_O	8.46	8.26	8.49	8.4	8.5	8.29	8.44	8.77	-	9.51	8.94
95	C_5_H_6_N_2_O_2_	8.81	8.62	8.88	8.82	8.89	8.71	8.87	9.19	-	9.08	9.2
96	C_4_H_4_N_2_O_2_	9.38	9.22	9.26	9.19	9.55	9.22	9.38	9.94	-	10.12	9.68
97	CH_4_N_2_O	9.58	9.33	9.6	9.4	9.59	9.32	9.46	9.94	-	10.05	9.8
98	Ag_2_	8.11	8.27	8.12	8.04	7.95	7.07	-	8.57	-	7.49	7.66
99	Cu_2_	7.24	7.66	-	-	7.4	7.54	7.78	8.6	-	7.57	7.46
100	NCCu	9.93	10.12	-	-	9.99	9.42	9.56	10.91	-	10.85	-

a Reference GW results from other codes are also provided (AIMS, TM, and BGW from ref 34 and WEST from ref 34), together with experimental data.^102^. For a full comparison with respect to the KIPZ results, we redirect the reader to ref 103.

**Table 4:** Quasiparticle Electron Affinity for the Selected Molecule of the GW100 Data Set as Obtained Within This Work, Compared with the Available Results of West_lin_^extra^ ^37^ ^,^ ^a^

index	formula	PBE (this work)	PBE (ref 37)	yambo_MPA_	yambo_PPA_	WEST_lin_
7	Li_2_	1.75	1.78	0.73	0.76	0.58
8	Na_2_	1.7	1.77	0.73	0.75	0.58
9	Na_4_	1.8	2.09	1.0	1.03	1.04
10	Na_6_	1.43	1.89	0.87	0.93	1.0
11	K_2_	1.33	1.6	0.72	0.79	0.72
12	Rb_2_	1.17	1.55	0.59	0.65	0.71
14	P_2_	3.42	3.42	1.16	1.08	1.08
15	As_2_	3.39	3.4	1.17	1.08	1.08
16	F_2_	5.94	5.94	0.7	0.32	1.05
17	Cl_2_	4.22	4.22	1.5	1.25	1.37
18	Br_2_	4.49	4.49	2.0	1.79	1.87
19	I_2_	4.44	4.45	3.15	3.18	3.21
26	C_4_	6.05	6.05	3.18	2.93	3.11
29	C_8_H_8_	2.29	2.31	0.22	–0.04	0.05
35	CCl_4_	2.69	2.7	0.55	0.31	0.39
36	CBr_4_	3.48	3.5	1.62	1.42	1.44
37	CI_4_	4.11	4.21	3.1	3.06	3.03
41	Si_5_H_1_2	1.22	1.68	–0.12	–0.17	0.05
42	LiH	1.59	1.58	0.1	0.1	0.05
43	KH	1.6	1.61	0.32	0.3	0.22
54	F_2_Mg	2.64	2.64	0.31	0.25	0.32
55	TiF_4_	4.07	4.08	0.7	0.29	0.82
56	AlF_3_	2.61	2.61	0.12	0.03	0.14
58	SF_4_	3.57	2.96	3.13	3.35	0.05
59	BrK	1.82	1.87	0.45	0.42	0.39
60	GaCl	2.39	2.39	0.46	0.45	0.42
61	NaCl	2.22	2.22	0.49	0.47	0.45
62	MgCl_2_	2.57	2.59	0.76	0.72	0.68
63	AlI_3_	2.58	2.82	1.57	1.54	1.65
66	PN	3.41	3.41	0.58	0.35	0.49
77	CS_2_	2.79	2.83	0.56	0.33	0.48
81	O_3_	6.17	6.17	2.56	1.87	2.6
82	SO_2_	4.4	4.42	1.37	1.02	1.36
83	BeO	4.84	4.84	2.25	2.27	2.23
84	MgO	4.29	4.29	1.95	1.71	2.03
93	C_5_H_6_N_2_O_2_	2.24	2.29	0.19	–0.11	0.08
94	C_4_H_4_N_2_O_2_	2.44	2.45	0.2	–0.05	0.13
96	Ag_2_	3.08	3.1	1.54	1.52	1.47
97	Cu_2_	3.09	3.1	1.3	1.3	1.29
98	NCCu	4.12	4.12	1.91	1.85	1.92

a Both DFT and QP values are indicated.

### 3.1 Ionization Potentials

We first examined the computed KS (PBE) HOMO energies, which serve as starting point for the subsequent *GW* QP corrections. These results are reported in Sec. II of the Supporting Information.^78^ The agreement with previous quantum espresso calculations^37^ is very good; the remaining small discrepancies arise primarily from the different supercell shape adopted in this work (see Sec. [Sec sec2.2]). Compared with results obtained using localized basis set codes, such as fhi-aims or tm, the differences reflect both the different basis representations and the use of PPs, which influence the high-energy part of the spectrum. Interestingly, the present PBE HOMO values agree well (within 40 meV on average) with def2-QZVP GTO results (see ref 34 and the related Supporting Information), a few meV better than the corresponding extrapolation to the complete basis-set limit.

Moving to the discussion of *GW* QP-corrections for the HOMO and LUMO states, the situation becomes more complex. The *GW* QP corrections for HOMO and LUMO levels depend on several numerical parameters. As detailed in Sec. [Sec sec2.5], we employed an extrapolation scheme with respect to the number of included empty states and the kinetic energy cutoff adopted to represent the response function. The accuracy of this procedure is analyzed in the Supporting Information.^78^ Overall, calculations done with the tightest convergence parameters are close to full convergence. This is particularly true for the IP computed within PPA, for which the Bruneval–Gonze terminator^67^ (available only for PPA) proved essential for accelerating convergence due to the dense manifolds of empty states in finite systems.

Next, we analyze the *G*_0_ *W*_0_ IPs obtained with the GN–PPA and MPA schemes of yambo. Table 2 summarizes the statistical indicators (ME, MAE, MARE, and σ) of yambo results with respect to other community codes. Beyond GW codes, we also include results from Koopmans spectral functionals (KIPZ)^103^ and quantum chemistry (CCSD)^101^ methods, in such a way as to also provide a brief comparison with different theoretical level/approximations. The GN–PPA data exhibit slightly larger deviations, as expected from the simpler frequency treatment of the PPA, while MPA results tend to show smaller σ, overall indicating improved accuracy. When compared with other PW or mixed-basis implementations, such as WEST, VASP, and fhi-aims, both approaches attain comparable accuracy.

The individual IPs of all molecules are reported in Table 3, together with previously published GW100 results,^34^ CCSD,^101^ and experimental data from the NIST database.^102^ The best agreement for the yambo GN–PPA results, as measured by the MAE, is obtained with WEST_sol_ and WEST_lin_, followed by VASP and fhi-aims in the complete-basis-set limit. This level of consistency is remarkable, given the methodological differences among these codes, including the choice of basis sets (plane waves, augmented waves, or localized orbitals), the use of PPs, the different ways to solve the QP equation (full or linearized), and the different frequency-integration schemes. Specifically, the frequency integration is performed using AC (vasp), CD (west), and MPA (yambo). Overall, these differences have only a minor effect on the computed QP energies, confirming the robustness of modern *G*_0_ *W*_0_ implementations.

In contrast, larger discrepancies arise when comparing the yambo GN–PPA results with those obtained using the HL-PPA model of bgw. Despite both adopting the linearized QP equation, the two models differ in their parametrization of the dielectric response: the HL and GN versions of PPA can yield significantly different QP corrections depending on the system and the treatment of the so-called unfulfilled modes.^17,26,45,46^ A semianalytical comparison between the two has shown that the HL–PPA may systematically overestimate QP shifts with respect to GN-PPA.^49^ Conversely, the FF bgw data show a noticeably better agreement with yambo, although the limited number of reported values (19 molecules) prevents a comprehensive comparison.

The yambo-MPA results generally improve the agreement with all other codes relative to GN–PPA, as reflected by the smaller MAE and MARE values in Table 2. The improvement is particularly significant when comparing with WEST_lin_ and VASP, confirming that MPA provides an efficient description of frequency dependence, with an accuracy comparable to that of FF-RA, CD, and AC approaches.^49,50,86^ The only notable exception is the comparison with fhi-aims results, based on localized def2-QZVP basis sets, prior to complete-basis-set extrapolation, for which slightly larger deviations persist. It is worth noting that, despite the absence of the Bruneval–Gonze terminator^67^ in the MPA implementation, which, at the PPA level, has been shown to accelerate convergence, the overall quality of the extrapolated MPA data remains unaffected. Finally, the relatively large MAE observed between the yambo GW data and experimental IPs can be attributed to several factors beyond the computational framework, including the neglect of finite-temperature effects and coupling with ionic degrees of freedom, as well as higher-order corrections such as self-consistency and vertex corrections.^34^ This is also consistent with the discrepancy observed with respect to the KIPZ method, which indeed can be considered as including approximate vertex corrections. For more details on the Koopmans functionals approach, we refer to refs 103–106 .

A more detailed assessment of the deviations across different implementations can be obtained from the violin plots in Figure 2, which combine box- and density-plot representations. The vertical asymmetry of each distribution indicates whether the IPs computed with yambo are systematically over- or underestimated relative to the reference data, while the width of the main peak reflects the standard deviation σ reported in Table 2. The values plotted in Figure 2 are summarized in Table 3, with PPA (yellow) and MPA (red) results shown side by side. For VASP, 16% (36%) of the molecules deviate by approximately −95 (30) meV for PPA (MPA), forming the largest peak in each respective distribution. When comparing yambo–PPA with WEST_lin_, 19% of the molecules cluster around −80 meV, while 32% form a shoulder at about −300 meV. In contrast, the MPA results show a narrower and more symmetric distribution, with 35% of the molecules deviating only by 19 meV from West_lin_, without any shoulder. Overall, yambo–MPA consistently improves the agreement with all other reference codes, further demonstrating the reliability of the MPA formulation.

**Figure 2:**
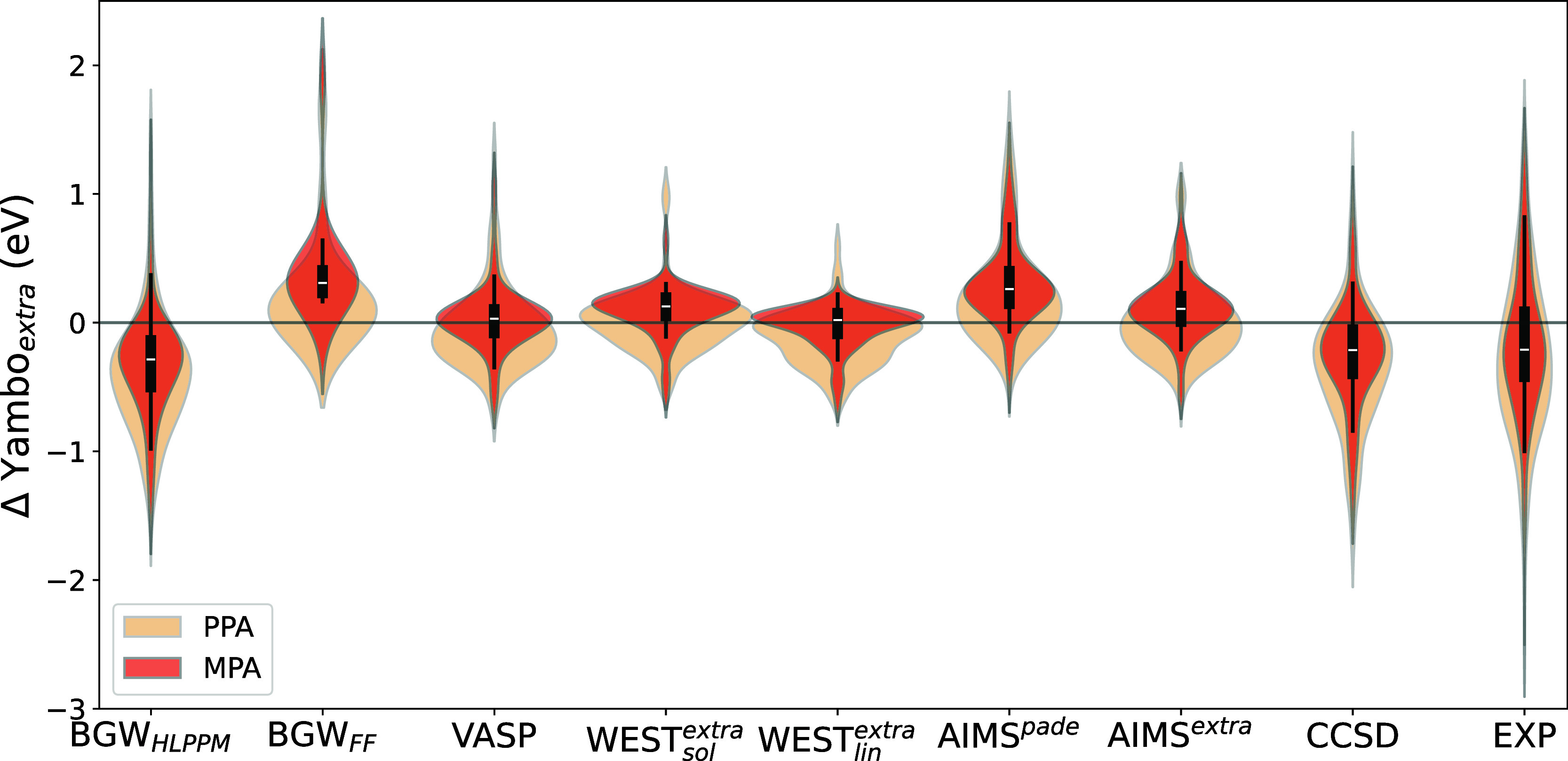
Violin plots representing the distribution of the IP deviation between yambo PPA and MPA results and other GW codes. The width of each curve reflects the density of data points. In the MPA case, the vertical black box represents the interquartile range, and the white horizontal line indicates the median.

Most distributions in Figure 2 are asymmetric toward a slight overestimation of the IPs computed with yambo relative to the other methods. The exceptions are the comparisons with BGW_HL‑PPA_, CCSD, and experimental data, where the deviation signs reverse. Outliers are present in almost all distributions. The largest deviation from VASP corresponds to the Xe molecule with differences of 1.30 eV (PPA) and 1.12 eV (MPA). For WEST_lin_, the largest outlier is the F_2_ molecule, with a PPA deviation of 633 meV, while other fluorine-containing molecules such as CF_4_, FH, and AlF_3_ show absolute deviations of 400–500 meV. These discrepancies are probably due to the presence of strongly localized 2p and 3d electrons, which are challenging to treat accurately in standard one-shot *G*_0_ *W*_0_ calculations,^107^ particularly within PPA.^26^ Importantly, the MPA approximation substantially mitigates these discrepancies, reducing, for example, the F_2_ difference to 273 meV. The improved performance of the MPA approach is further supported by the cumulative statistics: the number of molecules for which the difference between yambo and VASP (WEST_lin_) is smaller than 150 meV increases from 42 (50) in PPA to 72 (73) in MPA.

Finally, we briefly discuss the main factors influencing the residual discrepancies observed in the present data set: (*i*) the reduced finite size of the simulation cell, and (*ii*) the linearization of the QP equation. Regarding the supercell size, as discussed in Sec. II of the Supporting Information.^78^ the use of a 13 Å FCC cell—smaller than the 25 Å cubic cells employed in WEST^37^—introduces a finite-size error in the *G*_0_ *W*_0_ IPs. For molecules of average size, this amounts to roughly 25 meV, while for the largest molecules, it increases to about 180 meV. Consistently, when comparing with codes that use similar computational setups (e.g., WEST_lin_ or VASP), the MAEs lie within the expected uncertainty range.

Regarding the linearization of the QP equation, it shows a moderate impact when compared with WEST data obtained with and without linearization, changing the MAEs relative to yambo–PPA and MPA by about 20 and 60 meV, respectively. Finally, we did not find any significant correlation between the residual extrapolation error (with respect to the most converged yambo results) and the deviations from other codes, as shown in Figure S5 of the Supporting Information.^78^ This indicates that differences in the final IPs are only marginally influenced by the extrapolation procedure.

### 3.2 Electron Affinities

Electron affinities are significantly more difficult to compare than IPs. As noted in the original GW100 paper,^34^ the local orbital description of the unbound states, particularly for small molecules, is not well-converged, leading to large deviations with respect to results obtained with plane waves. For this reason, we compare the Yambo results only with the WEST code and the linearized *G*_0_ *W*_0_ QP solution, which showed the best agreement for the HOMO levels. The analysis of the EA energies follows the same protocol as for the IPs. A summary of the computed values is reported in Table 4, while Figure 3 displays the statistical differences between yambo and WEST. We first notice that the PBE results are in very good agreement with the corresponding results obtained in ref 37, showing a small MAE of 80 meV.

**Figure 3:**
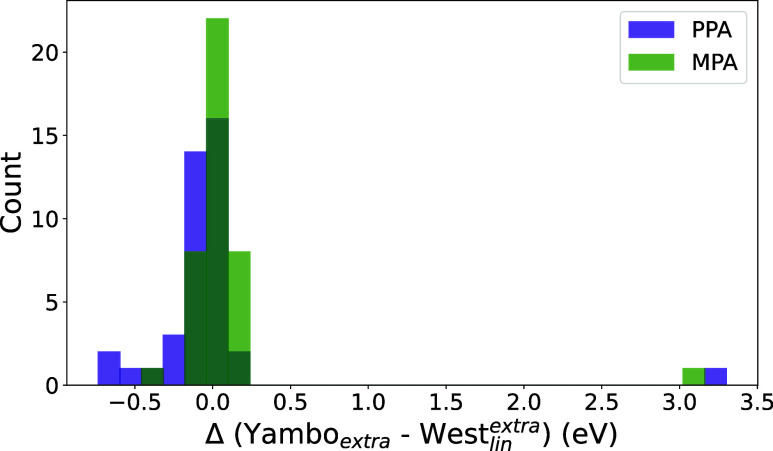
Deviation of the quasiparticle electron affinity (EA) between the yambo and the WEST linearized extrapolated results.

As observed for the IPs, MPA systematically improves the agreement with the reference data compared to that of PPA. The MAE amounts to 163 meV for MPA and 217 meV for PPA, confirming that the MPA representation yields a more accurate frequency dependence of the self-energy, including for unoccupied states. The largest deviation in the data set corresponds to the SF_4_ molecule, for which the LUMO exhibits an anomalously high discrepancy (approximately 600 meV) already at the DFT level.^37^ This deviation propagates to the QP correction and thus accounts for the outlier observed in Figure 3. Overall, the EA data set reinforces the conclusions drawn from the IP analysis: the MPA approach achieves accuracy comparable to that of FF methods at a substantially reduced computational cost. A comprehensive table of QP EA as obtained within this work is provided in the Supporting Information.^78^

## 4 Conclusions

In this work, we present a comprehensive benchmark of IPs and EAs for the 100 molecules of the GW100 data set, computed at the *G*_0_ *W*_0_ level with the yambo code. The study provides the first systematic assessment of the GN-PPA and the recently introduced MPA, within a PW PP framework. All calculations were performed using GPU-accelerated resources and automated workflows via the aiida-yambo plugin, ensuring reproducibility and high-throughput efficiency.

Overall, our results show very good agreement with reference data from other *GW* implementations, including VASP, WEST, BGW, and FHI-aims, with mean absolute errors of typically below 0.25 eV. The best agreement is found with WEST_lin_ and VASP, codes that also use the linearized solution of the QP equation and plane waves and PAW basis sets, respectively. On the other hand, the largest deviations, which are found for codes with localized basis sets (AIMS, Turbomole), remain moderate. Comparison with KIPZ, CCSD, and experimental IPs confirms trends previously reported for the GW100 set, with residual differences largely due to the lack of finite-temperature and electron–phonon effects, as well as the absence of self-consistency and vertex corrections in the present one-shot *G*_0_ *W*_0_ framework.

Importantly, the comparison between different frequency-integration schemes highlights the reliability of GN-PPA and its partial improvement over the Hybertsen–Louie PPA version used in BGW, which shows larger deviations with respect to full-frequency results. MPA further enhances the accuracy of the frequency treatment, providing good agreement with full-frequency approaches (analytic continuation or contour deformation) at a fraction of their computational cost. MPA extrapolated results also seem not to suffer from the lack of the BG terminator,^67^ used here with PPA.

The remaining discrepancies can be mainly attributed to the use of norm-conserving PPs and the finite size of the supercells. The linearization of the QP equation contributes only marginally (20–60 meV) to the differences with respect to other *GW* implementations. The error due to the extrapolation scheme with respect to the number of included empty states and the kinetic energy cutoff does not show a significant correlation with the deviations from other code results, suggesting that the extrapolation scheme is a valid method to improve accuracy with respect to the results obtained with the largest feasible convergence parameters.

In summary, the present benchmark demonstrates that GN-PPA and, in particular, MPA provide accurate and efficient treatments of *G*_0_ *W*_0_ QP calculations, enabling verification against more demanding full-frequency methods. These results consolidate the reliability of the yambo code for large-scale molecular *GW* simulations. Future developments will target the implementation of self-consistent *GW* and beyond-*GW* schemes on GPU architectures, further enhancing both the accuracy and the scalability of excited-state calculations.

## Supplementary Material



## Data Availability

The data presented in this work are available on the Materials Cloud Archive^108^ at the URL: 10.24435/materialscloud:4a-d7.

## Notes

Additional details on computational resource requirements, supercell choice, and the extrapolation procedure used to obtain the results of this work (PDF)
