# Supplementary material for: Benchmarking the Plasmon-Pole and Multipole Approximations in the Yambo Code Using the GW100 Data Set
Source: J Chem Theory Comput. 2026 Jun 12;22(13):6718–30. doi: 10.1021/acs.jctc.6c00239 (PMC13374005; doi:10.1021/acs.jctc.6c00239)
Supplement: Supplementary file 1 [file ct6c00239_si_001.pdf]

Benchmarking the plasmon-pole and multipole approximations in the Yambo Code using the GW100 dataset: Supplementary materials

M. Bonacci,[1,2,3] D. A. Leon,[4] N. Spallanzani,[1] E. Molinari,[1,5] D. Varsano,[1] A. Ferretti,[1] and C. Cardoso[1,*]

[1] *Istituto Nanoscienze – CNR, S3, Via G. Campi 213/A, 41125 Modena, Italy*
[2] *PSI Center for Scientific Computing, Theory and Data, 5232 Villigen PSI, Switzerland*
[3] *National Centre for Computational Design and Discovery of Novel Materials (MARVEL), 5232 Villigen PSI, Switzerland*
[4] *Department of Mechanical Engineering and Technology Management, Norwegian University of Life Sciences, NO-1432 Ås, Norway*
[5] *Dipartimento di Scienze Fisiche, Informatiche e Matematiche, Università di Modena e Reggio Emilia, I-41125 Modena, Italy*

I. COMPUTATIONAL DETAILS: RESOURCE REQUIREMENTS

In this Section we complement the computational details provided in the main text by discussing the HPC resources required to run the GW100 dataset with our computational setup. These include the memory requirements as well as the time-to-solution. Automating large numbers of simulations—particularly within the computationally demanding MBPT framework requires the use of high performance computing (HPC). As in the case of the present workflow, modern HPC architectures very often take advantage of hardware accelerators based on graphical processing units (GPUs), significantly reducing the time- and energy- to-solution with respect to CPU-only machines.

Concerning memory requirements, one of the largest pieces of allocated memory of our computational workflow originates from the representation of the response function as a dense $\chi_{\mathbf{GG}'}$ matrix. As discussed in Sec. II of the main text, this motivated us to adopt a cubic face centered (FCC) cell rather than a simple-cubic (SC), since the FCC geometry decreases the simulation volume without reducing the minimum distance between periodic images. In Fig. S.1 we show the scaling of the $\chi_{\mathbf{GG}'}$ matrix size with respect to the plane-wave (PW) cutoff G_{cut}, for an FCC cell with 13 Å side. The memory reaches 40 GB for $G_{cut} = 43$ Ry, while the memory required for $G_{cut} = 15$ Ry with an SC cell with 20 Å side is already 45 GB, exceeding the capabilities of the GPU used in the present work (NVIDIA A100 with 40 GB RAM.) One should also consider that this memory limitation is further increased by the storage of additional quantities such as wavefunctions or temporary workspace for linear algebra operations. This memory limitation has been overcome in recent versions of the YAMBO code by implementing an interface to distributed linear algebra solvers on GPUs (such as cuSOLVERMp from the NVIDIA software stack).

In the following, we discuss the timings and memory requirements for both PPA and MPA calculations. In Fig. S.1 we show the wall-time for a typical G_0W_0 flow (with frequency integration treated at the PPA level) performed on the Juwels-Booster cluster [1] (Jülich, Germany) using 20-64 nodes, each of them equipped with 4 A100 GPUs with 40 GB of memory. Each individual calculation takes less than 10 minutes to be completed, leading to a total of 1.5 hours per molecule, when considering 24 simulations. This performance depends on the significant speed-up provided by GPU-accelerated machines and codes.

On the basis of the above analysis, the predicted wall-time required to run the whole GW100 set at the PPA level was then estimated in about 150 hours.

In practice, the wall-time was reduced to ∼150/8 h = 18.75 hours, since on average eight calculations were running simultaneously and automatically, i.e. without human intervention to submit new jobs, thanks to the AiiDA scheduling system. In contrast, MPA calculations were computed using 20–64 nodes of the Leonardo supercomputer (Cineca, Italy), equipped with 4 NVIDIA A100 GPUs (64 GB of memory) per node. Simulations took ∼13 hours per molecule and a total time of 1100 hours (with an effective wall-time of about 140 hours). The

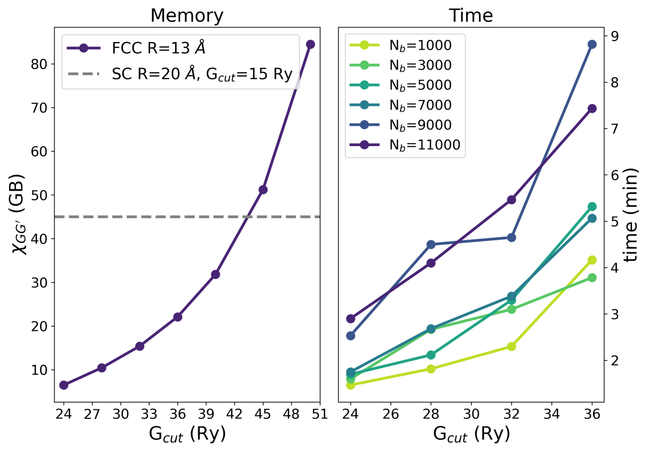

FIG. S.1: Left panel: memory needed to store the polarizability matrix $\chi_{GG'}$ with respect to the PW cutoff G_{cut}. Right panel: time-to-solution with respect to N_b and G_{cut} using GPU acceleration, for the PPA case.

*corresponding author:claudiamaria.pereiracardoso@cnr.it

MPA simulations are computationally more expensive than PPA, due to the more complex frequency treatment of the polarizability. Moreover, the number of bands used, N_b, is laso larger (on average, 2000 more empty states for each calculation), since the terminator technique [2], which accelerates convergence with respect to the summation over empty states, is not implemented for MPA.

II. SUPERCELL CHOICE

Calculations of low-dimensional systems using plane-wave codes, which implicitly assume periodic boundary conditions, require large unit cells to include sufficient vacuum and avoid spurious interactions between replicas. At fixed energy cutoff, larger cells require a larger number of plane-waves. Consequently, when computing the response function in reciprocal space, the size of the screening matrix scales quadratically with the number of PWs, or equivalently, with the cube of the kinetic energy cutoff G_{cut} and linearly with the volume of the cell (see Fig. S.1). For this reason, it is derirabe to keep the supercell as small as possible. Previous GW100 calculations [3] used a SC supercell with lattice parameters as large as 25 Å. In this work, we employ a n FCC cell while keeping the same distances between the nearest neighboring (NN) molecules. This choice reduces the supercell volume by a factor of $\sqrt{2}$ compared to the SC cell.

The DFT PBE HOMO energies are shown in Tables S.III and S.IV. Considering the reduced amount of vacuum used here, the DFT PBE values agree well with previously reported results [3]. In particular, the average discrepancy is 30 meV and ,for more than 74% of the molecules, the error smaller than 10 meV. Fig S.2 shows the differences between the HOMO energies obtained at the DFT/PBE and the G_0W_0 level, with the Yambo and WEST codes. There is a strong positive correlation, r=0.74 (Fig. S.2), in the differences obtained with Yambo MPA@G_0W_0 and those obtained with WEST 3. In contrast, for PPA@G_0W_0, the correlation is weak ($r = 0.33$), consistent with the simpler treatment of the frequency dependence with respect to MPA. Notably, the two deviation sets plotted in Fig S.2 share part of the outliers (for example the ones around 0.5-0.6 eV). This indicates that some of the outliers are mostly due to the different DFT starting points, and the agreement between the two codes can be improved by using a larger supercell. To estimate the effect of the supercell choice, we performed a statistical analysis extnede t othe other codes, similar to the one described in the main text for Table II, now considering only molecules with a discrepancy between YAMBO and WEST DFT results smaller than 5 meV (i.e. 64% of the molecules in the set). The results are summarized in Table S.I and show an improvement of only a few tens of meV, mainly for the comparison with $\text{WEST}^{extra}_{lin}$ and VASP. This demonstrates that while the DFT-level deviation due to the reduced supercell is measurable, its propagation to the G_0W_0 results has a limited impact on the statistical analysis and does not affect the overall conclusions presented in the main text.

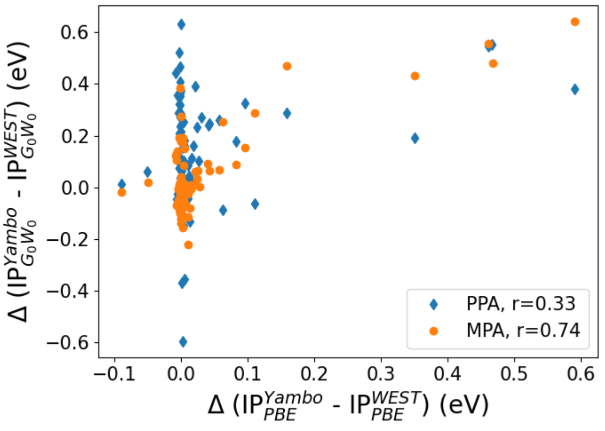

FIG. S.2: Plot of the deviation between the DFT PBE IP energies obtained in this work and in the WEST GW100 paper [3] versus the same deviation but for the G_0W_0 IP energies, for both the linearized and the secant solution obtained within the WEST code. In the legend, the correlation r between the G_0W_0 and DFT deviations is reported.

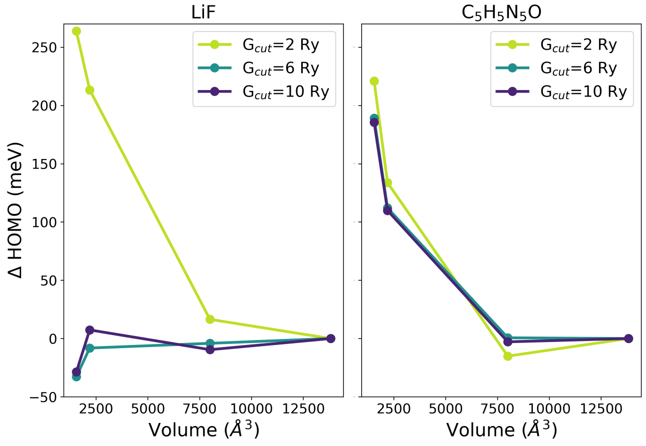

FIG. S.3: Convergence of the (PPA) HOMO level for two molecules, LiF and $C_5H_5N_5O$. The number of bands is kept fixed, N_b=0.75 Ry, while varying G_{cut} and the cell volume. The first point corresponds to an FCC cell with lattice **R**=13 Å. The other 3 points correspond to SC cells of lattice vector **R**=13, 20, and 24 Å. The results obtained for the maximum cell volume are set to zero for each fixed value of G_{cut}.

To provide a more direct estimate of the error in the quasiparticle calculations, we performed convergence tests with respect to the supercell volume for two representative molecules: LiF and $C_5H_5N_5O$ (see Fig. S.3). The first has 10, while the latter has 56 valence electrons and the largest linear size in the GW100 set, 7.5 Å. We considered a 13 Å FCC cell and three cubic cells with side lenghts 13, 20, and 24 Å while using under-converged N_b and G_{cut} values. The results are shown

Code	**ME** (meV)		**MAE** (meV)		**MARE** (%)		**σ** (meV)	
	GN-PPA	MPA	GN-PPA	MPA	GN-PPA	MPA	GN-PPA	MPA
$\mathrm{YAMBO}_{\text{GN-PPA}}$	-	103	-	168	-	1.6	-	116
$\mathrm{WEST}^{extra}_{lin}$	-91	11	166	86	1.5	0.8	164	68
$\mathrm{WEST}^{extra}_{sol}$	57	159	159	174	1.7	1.7	187	109
VASP	-22	80	210	126	2.0	1.2	209	188
AIMS^{pade}	239	342	308	351	3.3	3.5	307	244
AIMS^{extra}	59	174	213	196	2.2	2.0	224	186
$\mathrm{BGW}_{\text{HL-PPA}}$	-438	-335	516	395	4.2	3.3	364	331
BGW_{FF}	200	394	264	407	2.7	4.0	393	398
CCSD	-381	-273	459	355	3.8	3.0	374	317
EXP	-249	-142	525	456	4.4	3.9	449	436

TABLE S.I: Statistical errors between Yambo and other codes and experiments considering only a reduced set of the GW100 molecules, where the deviation between the DFT HOMO energy computed within the reduced supercell and the one of Ref. 3 is less than 5 meV.

in Fig. S.3. DFT calculations are performed using the Martyna-Tuckerman method [4], which cures spurious electrostatic effects due to the long-range Coulomb interaction. Instead, the *GW* calculations use a spherical truncation of the Coulomb potential with a diameter $\sim$ 1Å shorter than the lattice parameter. Notably, this Coulomb cutoff analytically removes the divergence of the long-range Coulomb interaction $v(\mathbf{q} \to 0)$ [5]. Numerically, we find that the results are converged within ~25 and ~180 meV for LiF and $C_5H_5N_5O$, respectively. Considering that all other molecules are smaller than $C_5H_5N_5O$ and that the final results are computed with larger convergence parameters, we estimate the error due to the use of a finite FCC cell to have an upper bound of 180 meV.

	MAE (meV)		σ (meV)	
	PPA	MPA	PPA	MPA
IP	68	199	25	95
EA	38	121	30	99
IP-EA	29	104	80	95

TABLE S.II: Mean absolute error (MAE), and standard deviation (σ) for the deviation from extrapolated and most converged quasiparticle HOMO-LUMO gaps.

III. THE EXTRAPOLATION PROCEDURE

We start by comparing IP and EA computed with the tightest convergence parameters with the corresponding extrapolated values, according to the procedure described in Sec. II of the main text. In Fig. S.4 we show an histogram of frequency distribution of the deviation between the two set of results, and in Tab. S.II we present the mean absolute error (MAE) and the standard deviation (σ) of the same data. For PPA, we have a MAE (σ) of 68 (25) and 38 (30) meV, respectively, for IP and EA. MPA shows considerably larger values of both MAE and σ, showing that the calculated results are further away from the extrapolated values. The MAE (σ) values for IP and EA are, respectively, 199 (95) and 121 (99) meV. This can be explained by the fact that MPA, contrary to PPA, does not take advantage of the terminator technique [2] that accelerates the convergence with respect to the summation over empty states. Notably, these deviations are still rather small since the mean absolute relative error (MARE) for the IP is only 0.65% (1.8%) for PPA (MPA).

We performed the same analysis regarding the evaluation of the HOMO-LUMO quasiparticle gap (Tab. S.II), which is expected to converge faster than the individual states. In fact, the MAE significantly decreases.

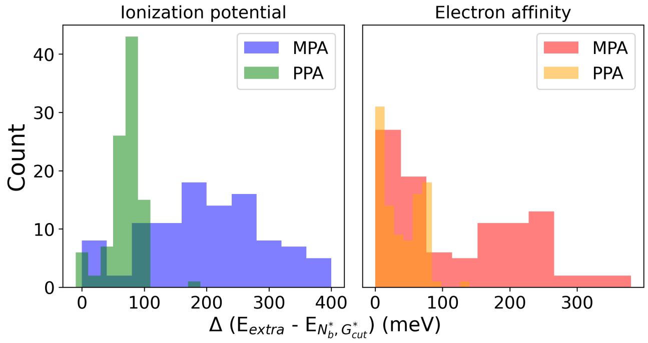

FIG. S.4: Convergence evaluation of YAMBO results for IP (left panel) and EA (right panel) with respect to the extrapolated value.

As an additional validation test for the extrapolation procedure, we computed the correlation between the error given by the extrapolation (Fig. S.4) and the discrepancy between Yambo results and other codes. Results, shown in Fig. S.5, indicate that there is no correlation between these two quantities, i.e. the extrapolation adds no relevant noise to the overall comparison.

[1] Jülich Supercomputing Centre JUWELS Cluster and Booster: Exascale Pathfinder with Modular Supercomputing Architecture at Juelich Supercomputing Centre. *Journal of large-scale research facilities* **2021**, *7*.

[2] Bruneval, F.; Gonze, X. Accurate G W self-energies in a plane-wave basis using only a few empty states: Towards large systems. *Phys. Rev. B* **2008**, *78*, 085125.

[3] Govoni, M.; Galli, G. GW100: Comparison of Methods and Accuracy of Results Obtained with the WEST Code. *J. Chem. Theory Comput.* **2018**, *14*, 1895–1909.

[4] Martyna, G. J.; Tuckerman, M. E. A reciprocal space based method for treating long range interactions in *ab initio* and force-field-based calculations in clusters. *J. Chem. Phys.* **1999**, *110*, 2810–2821.

[5] Rozzi, C. A.; Varsano, D.; Marini, A.; Gross, E. K. U.; Rubio, A. Exact Coulomb cutoff technique for supercell calculations. *Phys. Rev. B* **2006**, *73*, 205119.

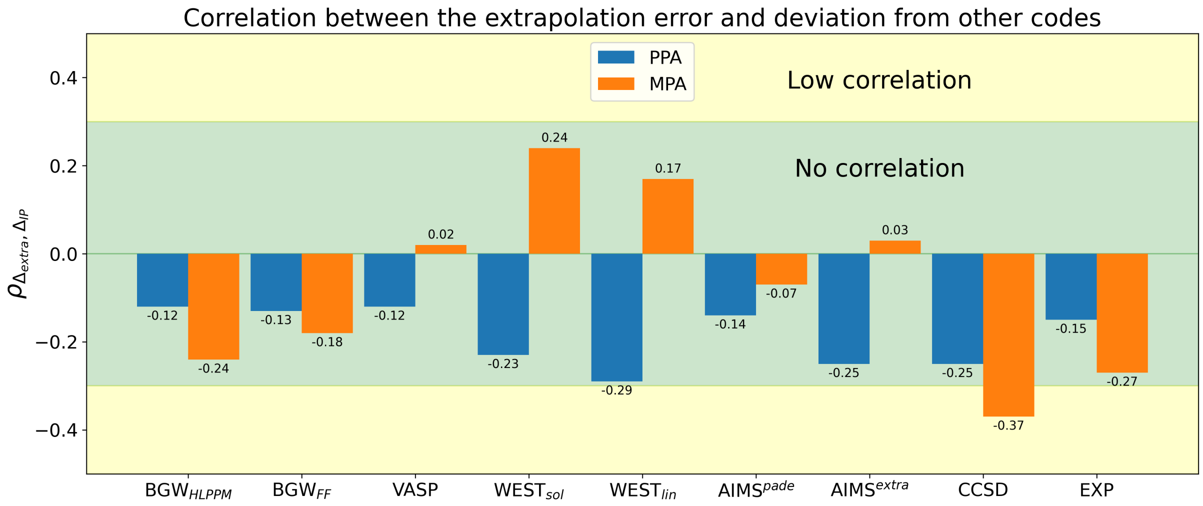

FIG. S.5: Correlation between the error given by the extrapolation procedure (Δ_{extra}) and the discrepancies with other codes (Δ_{IP}), indicated as $\rho_{\Delta_{extra},\Delta_{IP}}$. The correlation is considered to be significant only if larger than 0.5 (which is never the case for these results).

index	Formula	HOMO (PBE)	LUMO (PBE)
1	He	-15.76	-0.06
2	Ne	-13.33	-0.23
3	Ar	-10.26	-3.01
4	Kr	-9.25	-0.5
5	Xe	-8.23	-0.55
6	H_2	-10.38	-0.11
7	Li_2	-3.23	-1.75
8	Na_2	-3.12	-1.7
9	Na_4	-2.33	-1.8
10	Na_6	-2.4	-1.43
11	K_2	-2.51	-1.33
12	Rb_2	-2.37	-1.17
13	N_2	-10.29	-1.97
14	P_2	-7.11	-3.42
15	As_2	-6.49	-3.39
16	F_2	-9.42	-5.94
17	Cl_2	-7.28	-4.22
18	Br_2	-6.8	-4.49
19	I_2	-6.26	-4.44
20	CH_4	-9.46	-0.49
21	C_2H_6	-8.16	-0.52
22	C_3H_8	-7.76	-0.53
23	C_4H_10	-7.57	-0.5
24	C_2H_4	-6.77	-1.07
25	C_2H_2	-7.19	-0.42
26	C_4	-7.26	-6.05
27	C_3H_6	-7.06	-0.42
28	C_6H_6	-6.33	-1.24
29	C_8H_8	-5.28	-2.29
30	C_5H_6	-5.4	-1.48
31	C_2H_3F	-6.55	-0.98
32	C_2H_3Cl	-6.43	-1.42
33	C_2H_3Br	-5.83	-
34	C_2H_3I	-6.04	-1.69
35	CF_4	-10.43	-0.44
36	CCl_4	-7.66	-2.69
37	CBr_4	-6.92	-3.48
38	CI_4	-6.13	-4.11
39	SiH_4	-8.51	-0.55
40	GeH_4	-8.36	-0.79
41	Si_2H_6	-7.27	-0.7
42	Si_5H_12	-6.1	-1.22
43	LiH	-4.36	-1.59
44	KH	-3.48	-1.6
45	BH_3	-8.5	-2.95
46	B_2H_6	-7.88	-2.03
47	NH_3	-6.16	-0.79
48	HN_3	-6.82	-2.12
49	PH_3	-6.72	-0.72
50	AsH_3	-6.78	-0.87

TABLE S.III: DFT PBE HOMO and LUMO results as computed in this work.

index	Formula	HOMO (PBE)	LUMO (PBE)
51	SH_2	-6.29	-0.93
52	FH	-9.65	-0.99
53	ClH	-8.03	-1.12
54	LiF	-6.13	-1.5
55	F_2Mg	-8.31	-2.64
56	TiF_4	-10.45	-4.07
57	AlF_3	-9.72	-2.61
58	BF	-6.78	-2.11
59	SF_4	-8.37	-3.57
60	BrK	-4.72	-1.82
61	GaCl	-6.58	-2.39
62	NaCl	-5.29	-2.22
63	$MgCl_2$	-7.61	-2.57
64	AlI_3	-6.48	-2.58
65	BN	-7.47	-7.28
66	NCH	-9.04	-1.11
67	PN	-7.74	-3.41
68	H_2NNH_2	-5.28	-0.98
69	H_2CO	-6.27	-2.71
70	CH_4O	-6.34	-0.69
71	C_2H_6O	-6.16	-0.69
72	C_2H_4O	-5.97	-2.15
73	C_4H_10O	-5.77	-0.48
74	CH_2O_2	-6.95	-1.57
75	HOOH	-6.45	-1.73
76	H_2O	-7.25	-0.97
77	CO_2	-9.1	-0.96
78	CS_2	-6.79	-2.79
79	OCS	-7.48	-1.9
80	OCSe	-6.94	-2.05
81	CO	-9.34	-3.34
82	O_3	-7.95	-6.17
83	SO_2	-8.04	-4.4
84	BeO	-6.16	-4.84
85	MgO	-4.82	-4.29
86	C_7H_8	-5.97	-1.19
87	C_8H_10	-5.92	-1.08
88	C_6F_6	-6.64	-2.18
89	C_6H_5OH	-5.62	-
90	$C_6H_5NH_2$	-5.0	-1.09
91	C_5H_5N	-5.92	-1.9
92	$C_5H_5N_5O$	-5.21	-1.32
93	$C_5H_5N_5O$	-5.49	-1.67
94	$C_4H_5N_3O$	-5.71	-2.05
95	$C_5H_6N_2O_2$	-6.0	-2.24
96	$C_4H_4N_2O_2$	-6.27	-2.44
97	CH_4N_2O	-5.93	-1.01
98	Ag_2	-5.19	-3.08
99	Cu_2	-4.74	-3.09
100	NCCu	-6.78	-4.12

TABLE S.IV: Continuation of Table S.III.

index	Formula	Y_{MPA}	Y_{PPA}
1	He	-0.1	-0.19
2	Ne	-0.42	-0.43
3	Ar	-1.67	-1.71
4	Kr	-0.49	-0.49
5	Xe	-0.43	-0.45
6	H_2	-0.15	0.79
7	Li_2	0.73	0.76
8	Na_2	0.73	0.75
9	Na_4	1.0	1.03
10	Na_6	0.87	0.93
11	K_2	0.72	0.79
12	Rb_2	0.59	0.65
13	N_2	-2.11	-2.43
14	P_2	1.16	1.08
15	As_2	1.17	1.08
16	F_2	0.7	0.32
17	Cl_2	1.5	1.25
18	Br_2	2.0	1.79
19	I_2	3.15	3.18
20	CH_4	-0.49	-0.53
21	C_2H_6	-0.54	-0.55
22	C_3H_8	-0.53	-0.55
23	C_4H_10	-0.54	-0.57
24	C_2H_4	-1.72	-1.92
25	C_2H_2	-2.43	-2.59
26	C_4	3.18	2.93
27	C_3H_6	-0.54	-0.56
28	C_6H_6	-0.8	-1.02
29	C_8H_8	0.22	-0.04
30	C_5H_6	-0.79	-1.02
31	C_2H_3F	-1.81	-2.05
32	C_2H_3Cl	-1.12	-1.34
33	C_2H_3I	-0.14	-0.31
34	CF_4	-0.73	-0.75
35	CCl_4	0.55	0.31
36	CBr_4	1.62	1.42
37	CI_4	3.1	3.06
38	SiH_4	-0.52	-0.52
39	GeH_4	-0.45	-0.51
40	Si_2H_6	-0.6	-0.6
41	Si_5H_{12}	-0.12	-0.17
42	LiH	0.1	0.1
43	KH	0.32	0.3
44	BH_3	0.02	-0.02
45	B_2H_6	-0.66	-0.75
46	NH_3	-0.62	-0.65
47	HN_3	-1.09	-1.45
48	PH_3	-0.49	-0.5
49	AsH_3	-0.5	-0.51
50	SH_2	-0.59	-0.61

TABLE S.V: Quasiparticle electron affinities results as computed within this work.

index	Formula	HOMO (PBE)	LUMO (PBE)
51	FH	-1.04	-1.08
52	ClH	-0.98	-1.04
53	LiF	-0.05	-0.06
54	F_2Mg	0.31	0.25
55	TiF_4	0.7	0.29
56	AlF_3	0.12	0.03
57	BF	-0.93	-1.02
58	SF_4	3.13	3.35
59	BrK	0.45	0.42
60	GaCl	0.46	0.45
61	NaCl	0.49	0.47
62	$MgCl_2$	0.76	0.72
63	AlI_3	1.57	1.54
64	BN	4.02	3.79
65	NCH	-2.2	-2.43
66	PN	0.58	0.35
67	H_2NNH_2	-0.6	-0.64
68	H_2CO	-0.75	-1.06
69	CH_4O	-0.73	-0.75
70	C_2H_6O	-0.67	-0.7
71	C_2H_4O	-0.86	-1.2
72	$C_4H_{10}O$	-0.55	-0.58
73	CH_2O_2	-1.7	-1.95
74	HOOH	-1.85	-2.24
75	H_2O	-0.8	-0.83
76	CO_2	-0.88	-0.93
77	CS_2	0.56	0.33
78	OCS	-0.93	-1.21
79	OCSe	-0.75	-1.04
80	CO	-0.43	-0.68
81	O_3	2.56	1.87
82	SO_2	1.37	1.02
83	BeO	2.25	2.27
84	MgO	1.95	1.71
85	C_7H_8	-0.77	-0.94
86	C_8H_{10}	-0.8	-1.01
87	C_6F_6	-0.07	-0.24
88	$C_6H_5NH_2$	-0.85	-1.07
89	C_5H_5N	-0.25	-0.52
90	$C_5H_5N_5O$	-0.47	-0.77
91	$C_5H_5N_5O$	-0.16	-0.44
92	$C_4H_5N_3O$	0.04	-0.27
93	$C_5H_6N_2O_2$	0.19	-0.11
94	$C_4H_4N_2O_2$	0.2	-0.05
95	CH_4N_2O	-0.43	-0.46
96	Ag_2	1.54	1.52
97	Cu_2	1.3	1.3
98	NCCu	1.91	1.85

TABLE S.VI: Continuation of Table S.V.
